# Formulation and Characterization of PLGA Minocycline Microneedles for Enhanced Skin Deposition and Antibacterial Activity in Acne Treatment

**DOI:** 10.3390/polym17212912

**Published:** 2025-10-31

**Authors:** Juhaina M. Abu Ershaid, Suha M. Abudoleh, Dima N. Lafi, Nisreen A. Dahshan

**Affiliations:** 1 Department of Applied Pharmaceutical Sciences and Clinical Pharmacy, Faculty of Pharmacy, Isra University, Amman 11622, Jordan; ad0450@iu.edu.jo (D.N.L.); nisreen.dahshan@iu.edu.jo (N.A.D.); 2 Department of Basic Pharmaceutical Sciences, Faculty of Pharmacy, Middle East University, Amman 11831, Jordan; sabudoleh@meu.edu.jo

**Keywords:** acne vulgaris, minocycline, microneedles, PLGA, transdermal therapy, targeted therapy

## Abstract

Acne is a multifactorial skin condition characterized by an infection in the *pilosebaceous* units in the skin. Patients with acne suffer from comedones, papules, pustules and nodules or cysts in severe cases. These clinical features might cause disfigurmentation, depression, anxiety and significantly impact the quality of life of patients. Systemic and continuous exposure of antibiotics put patients at risk of developing systemic toxicity, bacterial resistance and gut dysbiosis. Microneedles offer an innovative approach of providing targeted topical delivery of minocycline while insuring efficient permeation through skin layers. **Methods:** minocycline microneedles were formulated using casting method and characterized for insertion ability, mechanical strength, drug content, antibacterial activities, deposition and dissolution behavior using ex vivo full-thickness rat skin. **Results:** Insertion tests confirmed effective skin penetration and mechanical integrity with only 9.5% height reduction. Drug content was 673.06 ± 5.34 µg/array. Dissolution occurred within 2 min in skin, indicating user-friendly wear time. Ex vivo Franz diffusion studies showed 26% of the drug deposited into the skin, significantly higher (*p* = 0.0068) than the 18.3% that permeated through it. Antibacterial testing revealed strong activity against *S. aureus*, *S. epidermidis*, and *C. acnes*, with MIC values < 0.146 µg/mL and MBC values ranging from 9.375–18.75 µg/mL. **Conclusions:** The result of this research demonstrate that minocycline microneedles effectively deliver minocycline into the skin highlighting their potential as a safer and more efficient alternative for acne therapy.

## 1. Introduction

Acne vulgaris is a chronic skin infection characterized by high sebum secretion, keratosis around hair follicles, inflammation and imbalance in androgen levels [1]. Ages between 12 to 25 years are more susceptible to this skin condition. Several factors play a major role in acne, including immunity, genetics, diet and stress [2]. Acne vulgaris causes permanent scars or skin pigmentation in case of improper treatment [3]. Oral or topical isotretinoin, contraceptives and antibiotics are used for treating acne [4]. Minocycline (MNC) is one of the widely used tetracyclines for this purpose, it inhibits the synthesis of proteins in bacterial ribosomes [5]. This class of antibiotics works on gram positive and gram-negative bacteria [6]. Commonly, MNC is prescribed daily for several months for acne vulgaris. MNC has both antimicrobial and anti-inflammatory affects which enhances therapeutic outcomes for skin conditions [7]. Oral MNC is highly distributed into the body fluids; therapeutic concentrations were detected in the cerebrospinal fluid upon oral administration [8].

Oral MNC has a relatively high percentage of side effects compared to other oral tetracyclines, 13.6% of the patients might develop side effects [9,10]. Oral antibiotics have a reduced bioavailability, around 50% of MNC is metabolized by the first pass metabolism in the liver [11].

**Figure 1:**
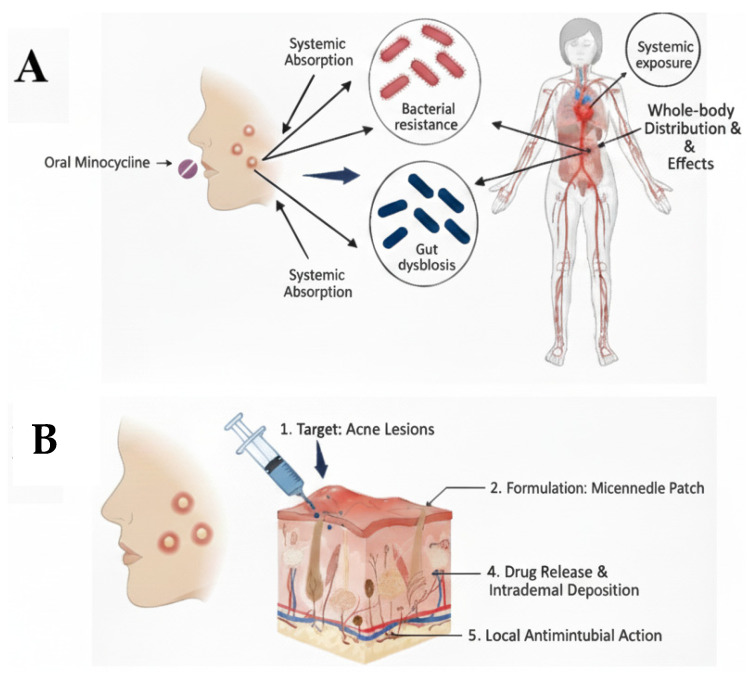
(**A**). Graphical illustration showing the consequences of treating acne vulgaris using oral minocycline, including bacterial resistance, gut dysbiosis and systemic exposure. (**B**). Graphical illustration showing the proposed project of developing a MNs transdermal patch loaded with minocycline to provide a sustained topical delivery and minimise the adverse effects associated with oral delivery.

Systemic recurrent exposure of MNC raises the concerns of developing bacterial resistance and normal flora imbalance as detailed in Figure 1. Unbalanced gastrointestinal microorganisms induce gut dysbiosis [12]. This condition increases the bacterial resistance and infections susceptibility, besides reducing the efficacy of the immunity system [13]. Moreover, concerns of developing fungal infections due to the broad activity of MNC are considerable [14]. Not to mention the other side effects of MNC affecting the cutaneous, genitourinary, respiratory, nervous and other systems. The oral administration of antibiotics such as MNC increases the risk of drug-drug or drug-food interactions which might lead to adverse effect, increase or decrease the efficacy of other medications [15,16]. There is a continuous effort from researchers to develop and investigate novel delivery systems for providing targeted antibiotics delivery including applying nanotechnology or innovative delivery systems [17,18,19].

Topical MNC was introduced to overcome the previously mentioned concerns with the systemic formulations. This form of MNC is available in the United States market as a topical foam [7]. On the other hand, the available topical MNC formulations are semisolid, this physical form of medications has a considerable stability concern which might affect the efficacy. Additionally, topical MNC must penetrate skin layers to reach the pilosebaceous unit where bacteria colonize [20].

Microneedles (MNs) technology is a novel transdermal drug delivery system; it composed of a set of 200–900 needles in the micro size supported by a baseplate [21]. This delivery system is designed to be applied on the skin, penetrate the outermost layer of the skin and eventually deliver therapeutics locally or to the systemic circulation without any pain [22]. There are four types of MNs, namely solid, polymeric, coated and hollow. These types differ in their design, composition, drug delivery mechanism and their applications [23]. Polymeric MNs include biodegradable, dissolving and hydrogel forming. Numerous studies showed the effective use of polymeric MNs in wide applications highlighting their ability to effectively penetrate the *stratum corneum* while ensuring painless and minimally invasive drug delivery [24]. Biodegradable MNs are made of polymers that biodegrade into basic molecules which the body can metabolize or excrete [25]. Poly (lactic-co-glycolic acid) (PLGA) is an intensively used biocompatible and biodegradable polymer for MNs formulations. Food and Drug Administration (FDA) approved this polymer owing to the high safety profile and degradable nature. PLGA degrade into lactic and glycolic acids which are excreted via the kidney [26].

PLGA MNs showed a great promise for transdermal drug delivery with various applications including protein delivery, vaccination and dermatological therapy [21,27]. PLGA MNs demonstrated their ability to encapsulate therapeutics while having sufficient mechanical properties to penetrate the *stratum corneum* for efficient delivery [27]. MNs array are composed of two main parts; the MNs and the baseplate. Loading the drug into the MNs only avoids drug wastage as they will be inserted into the skin leaving the baseplate free of drug. This design of PLGA MNs was previously investigated and showed effective drug delivery [26].

Applying this technology to deliver MNC topically reduces the systemic exposure and bacterial resistance without affecting the normal flora in the gastrointestinal tract. Also, avoids the problematic adverse effects of this drug and enhances the bioavailability due to eliminating the first pass metabolism [27,28,29,30].

At the same time, MNs technology enhances the physical stability of the drug, as their final physical form is solid. The microsized needles of this technology bypass the physical barrier of the skin and highly enhances the skin penetration [30,31].

Currently, there are a few published studies reporting minocycline microneedles for oral gingiva [32,33]. These studies showed the successful formation of minocycline microneedles using different polymers while illustrating the ability to deliver minocycline to treat periodontitis. Our study is a novel research introducing the first PLGA based minocycline microneedles for acne vulgaris. Our MNs demonstrated the mechanical length of PLGA for efficient skin penetration and achieved superior drug deposition in the skin representing a significant advancement in local drug delivery.

This study aims to develop poly lactic-co-glycolic acid (PLGA) tipped MNs loaded with MNC to provide a targeted delivery into the site of infection.

## 2. Materials

MNC was purchased from (Apollo Scientific, Manchester, UK). Dimethyl sulfoxide (DMSO) and phosphate buffer solution (PBS) were obtained from (Sigma Aldrich, St. Louis, MO, USA). Poly (D,L-lactide-co-glycolide), lactide: glycolide (50:50), average MW 10,000 to 15,000 was purchased from (Biosynth^®^ Ltd., Compton, UK). Poly (vinyl alcohol), 98.0–98.8% hydrolyzed, M.W. approx. 31,000–50,000 from (Thermo Fisher Scientific, Waltham, MA, USA). PVP (M.W.~58,000) from (GlpBio, Montclair, CA, USA). MNs moulds (8 mm × 8 mm, 8 mm × 8 mm patch, 10 × 10 array, Needle Height: 700, Needle Base: 200, Needle Pitch: 500) from (Amerigo Scientific, Hauppauge, NY 11788, USA). Nutrient broth and agar from (OxoidTM, Basingstoke, UK). Brain heart infusion broth and agar (Biolab, Budapest, Hungary) were used. Staphylococcus aureus (ATCC 9144), Staphylococcus epidermidis (ATCC 51625), and Cutibacterium acnes (ATCC 11827) were used. Rat skin was obtained from Isra University animal house (11-35/2022/2023).

## 3. Methods

### 3.1. PLGA-Minocycline Tipped Microneedles Fabrication

**Figure 2:**
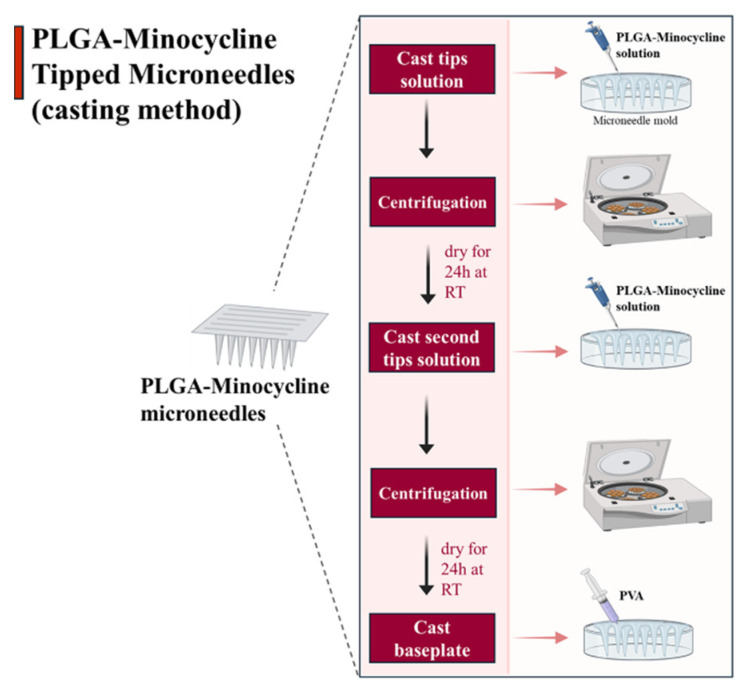
PLGA-minocycline tipped microneedles (MNs) preparation method. Tips solution was made of dissolving PLGA (35 mg/mL) and minocycline (25 mg/mL) in DMSO. This solution was used to formulate the tips of the MNs where 40 µL was added using micropipette into MNs moulds. Moulds were centrifuged for 15 min at 4500 rpm to concentrate the solution into the tips. Moulds were left to dry for 24 h at room temperature (RT) then another cast of tips solution with the same volume was performed followed by centrifugation. Moulds were left to dry for 24 h, then an aqueous blend of baseplate formulated from (PVA:PVP 20/20% *w*/*w*) was added using 3 mL syringe. Moulds were left to dry for 24 h at RT.

MNs were formulated using casting method where drug was only loaded into the tips of the microneedle’s moulds [21]. Firstly, tips solution was made of dissolving PLGA (35 mg/mL) and MNC (25 mg/mL) in DMSO. This solution was used to formulate the tips of the MNs where 40 µL was added using micropipette into MNs moulds. Moulds were centrifuged for 15 min at 4500 rpm to concentrate the solution into the tips. Moulds were left to dry for 24 h at room temperature (RT) then another cast of tips solution with the same volume was performed followed by centrifugation. Moulds were left to dry for 24 h, then an aqueous blend of baseplate formulated from (PVA:PVP 20/20% *w*/*w*) was added using 3 mL syringe. Moulds were left to dry for 24 h at RT as illustrated below in Figure 2.

### 3.2. PLGA-Minocycline Tipped Microneedles Characterization

PLGA-MNC MNs were characterized in terms of their insertion ability. MNs were inserted into full thickness rat skin using thumb pressure for 30 s. To visualize the created microholes by MNs, inverted microscope was used to visualize rat skin after MNs application. Additionally. Parafilm^®^ M Insertion test was conducted to evaluate the insertion ability of the prepared MNs. MNs were attached using adhesive paste to a movable probe of Agrosta^®^ Belle Texture Analyzer (Serqueux, France) and directed toward eight layers of folded Parafilm^®^ M placed on top of flat surface. The movable probe was moving in axial direction towards the Parafilm^®^ M layers in 0.5 mm/s speed and applied 32 N force for 30 s against MNs. Parafilm^®^ M layers were visualized using inverted microscope to determine number of holes per layer and the percentage of created holes was calculated according to Equation (1) [34]. Further, the mechanical evaluation of MNs was performed using Agrosta^®^ Texture Analyzer. MNs were attached using adhesive paste into a movable probe and directed towards a flat surface. Movable probe was programmed in compression mode and speed was set at 0.5 mm/s, 32 N was applied on MNs for 30 s. After performing this test, MNs were also visualize under inverted microscope to determine shape and lengths of MNs [35]. Percentage of lengths reduction was calculated according to the below Equation (2) [36].

(1) $$Created\;holes\%=\frac{Number\;of\;holes\;in\;each\;{Parafilm}^{®}M\;layer}{Total\;number\;of\;MNs\;}\times 100\%\;$$

(2) $$Height\;reduction\%=\frac{Hb-Ha}{Hb}\times 100\%$$

### 3.3. PLGA-Minocycline Tipped Microneedles Drug Content

MNC content in PLGA-MNC e tipped MNs was determined using UV-Spectrophotometer. MNs were dissolved in PBS and sonicated until full dissolution. Samples were further diluted and analysed using UV-spectrophotometer. MNC calibration curve was obtained before sample analysis over the concentration gradient (3.125–50 µg/mL) at wavelength (347 nm).

### 3.4. PLGA-MNC Tipped Microneedles In Vitro and Ex Vivo Dissolution Time

The in vitro dissolution time of MNs was determined by dissolving MNs in 20 mL of PBS under continuous stirring. MNs were observed until full dissolution and time was recorded. For ex vivo dissolution rate, MNs were inserted into rat skin and removed after (30, 60 and 120 s). At each timepoint, MNs were removed and visualized under inverted microscope.

### 3.5. PLGA-Minocycline Tipped Microneedles Ex Vivo Skin Deposition Study

Ex vivo skin deposition study was conducted using Franz diffusion cell apparatus. This experiment was performed to evaluate the ability of MNs to locally deliver vancomycin into full-thickness rat skin [37,38]. UV-spectrophotometer was used to quantify MNC in the reservoir compartment of the Franz cells and in rat skin. Rat skin was obtained from Isra University animal house. Skin was cleaned, shaved and rinsed with PBS before being stored at −70 °C using deep freezer. PBS (pH = 7.4) was used as the media of the reservoir compartment due to the hydrophilic nature of MNC. Skin pieces were attached to the donor compartment of Franz cells using metal clips. MNs were inserted into the center of the skin and gently pressed with fingers for 30 s using thumb. MNs were fixed using a cylindrical metal (diameter of 11.0 mm and a weight of 5.0 g). One layer of Parafilm^®^ M was placed on top of the donor chamber and receptor arm to prevent fluid evaporation. Temperature was controlled at 37 ± 1 °C during the experiment. The permeated amount of MNC into the reservoir compartment at predetermined time points (1, 3, 6 and 24 h) was determined. In details, one mL sample from reservoir champer was taken from the sampling arm then diluted with 2 mL of PBS and analysed using UV-spectrophotometer. Exact sample volume was replaced with fresh release media to maintain sink condition. After 24 h, skin pieces were removed from the Franz cell compartment, the remaining undissolved parts of MNs were discarded. Skin pieces were placed in 15 mL falcon tubes after cutting into tiny pieces using scissor. Skin pieces were immersed in 5 mL PBS and sonicated for 30 min. Then samples were centrifuged and supernatant was analysed using UV-spectrophotometer.

### 3.6. PLGA-Minocycline Tipped Microneedles Antibacterial Potency

#### 3.6.1. Agar Diffusion Method

The antibacterial activity of PLGA-MNC tipped MNs was tested using the agar diffusion method according to a previously prescribed method of with some modifications and the activity was compared with control microneedle loaded with PLGA and DMSO. The activity was tested against *S. aureus* (ATCC 9144), *S. epidermidis* (ATCC 51625), and *C. acnes* (ATCC 11827). First of all, Bacterial concentration was adjusted to 0.5 McFarland [39,40]. After that 100 µL of each bacterial preparation was equally spread over the surface of nutrient agar for *S. aureus* and *S. epidermidis*, and brain heart infusion agar (BHI) was used for *C. acnes*.

Then, the PLGA-MNC tipped MNes were gently inserted into the agar plate by sterile forceps. The plates were incubated at 37 °C under aerobic conditions for Staphylococci species and strict anaerobic conditions for *C. acnes* for 24 h. Finally, the diameter of inhibition zones surrounding the inserted microneedles was measured.

#### 3.6.2. Minimum Inhibitory Concentration (MIC)

The MIC was determined according to a previously described method using a 96-well plate assay [41]. In brief, 100 µL of nutrient broth was added to each well for *S. aureus* and *S. epidermidis*, while BHI broth was used for *C. acnes*. Then, 100 µL of the microneedle solution (MNC-PLGA-DMSO), MNC alone and PLGA-DMSO were separately added to the first well and serially diluted as the following: 300 µg/mL, 150 µg/mL, 75 µg/mL, 37.5 µg/mL, 18.75 µg/mL, 9.375 µg/mL, 4.687 µg/mL, 2.343 µg/mL, 1.171 µg/mL, 0.585 µg/mL, 0.292 µg/mL, and 0.146 µg/mL. Then, 100 µL of bacterial suspension (1 × 106 CFU/mL) was added to each well. The plates were incubated at 37 °C for 18 h. The MIC was recorded as the lowest concentration at which no visible bacterial growth was observed.

#### 3.6.3. Minimum Bactericidal Concentration (MBC)

The MBC is determined by sub-culturing 15 µL of MIC broth wells with no visible growth onto nutrient agar plates under aerobic conditions for Staphylococci species and BHI agar plate for *C. acnes*. The MBC was defined as the lowest concentration that required to kill the bacteria under defined conditions [42,43].

### 3.7. Statistical Analysis

The statistical analysis in this work was performed via GraphPad^®^ PrismV10 software. The data were processed via Microsoft^®^ Excel^®^ 2016. Independent sample *t* and ANOVA tests were used for comparing groups, and the significance level was set at *p* < 0.05.

## 4. Results

### 4.1. PLGA-Minocycline Tipped Microneedles Fabrication

The reported preparation method of PLGA-MNC MNs in this work successfully yielded fully formed MNs. Upon microscopic inspection, MNs had complete structural integrity with uniform lengths and without air bubbles.

### 4.2. PLGA-Minocycline Tipped Microneedles Characterisation

**Figure 3:**
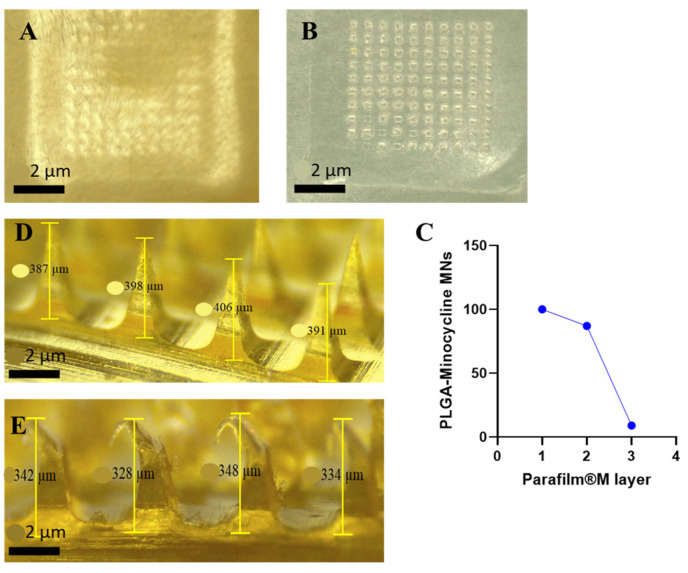
(**A**). Full thickness rat skin after MNs insertion using manual pressure by thumb pressing for 30 s. (**B**). Parafilm^®^ M after MNs insertion using Agrosta^®^ Texture Analyzer for 30 s and 32 N. (**C**). Insertion profile of MNs into Parafilm^®^ M layers using. (**D**). Microscope images of microneedles before compression force test using Agrosta^®^ Texture Analyzer for 30 s and 32 N. (**E**). Microscope images of microneedles after compression force test using Agrosta^®^ Texture Analyzer for 30 s and 32 N.

**Figure 4:**
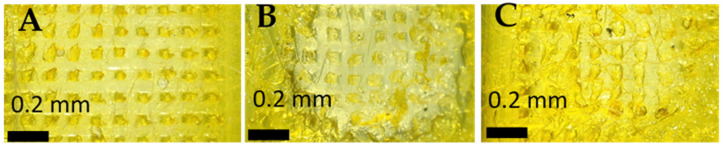
Ex vivo skin dissolution study of PLGA-minocycline tipped MNs into full thickness rat skin. (**A**). After 3 s of application. (**B**). After 60 s of application. (**C**). After 120 s of application.

MNs technology is applied in this research to overcome the physical barrier of the skin and provide efficient topical delivery of MNC. In order to achieve this purpose, MNs should be able to penetrate the outermost layer of the skin (*Stratum Corneum*) without mechanical failure [21]. MNs were evaluated in terms of their insertion ability manually on a full thickness rat skin and using Agrosta^®^ Texture Analyzer on Parafilm^®^ M. As shown in Figure 3, MNs were able to penetrate the skin where the created microholes by MNs were visualized on top of the skin. For Parafilm^®^ M insertion test, MNs were able to reach the second layer while they completely penetrated the first layer by a percentage of 100%. Each layer of Parafilm^®^ M has a thickness of 126 µm while thickness of stratum corneum varies between 10 to 20 µm [44]. Penetrating the second layer of Parafilm^®^ M indicates a total of 252 µm insertion depth which supports the ability of the prepared MNs to bypass the stratum corneum. Figure 4 details the percentage of created holes in each layer. In terms of the mechanical properties, MNs were subjected to compression force test to investigate the hardness of these MNs upon insertion. MNs were remained without mechanical failure during this test and all the performed tests. Percentage of heights reduction was calculated according to Equation (2). MNs demonstrated a height reduction percentage of 9.5 ± 5.6% where MNs had an average length of 0.38 ± 0.03 µm before compression test and 0.34 ± 0.02 µm after compression test. This finding is in parallel of previously reported studies demonstrating the high potential of MNs to be successfully inserted into skin in vivo [37,38].

### 4.3. PLGA-Minocycline Tipped Microneedles Drug Content

MNC was quantified in MNs using UV-spectrophotometer at 347 nm. Calibration curve was linear over the studied concentrations (3.125–50 µg/mL) with R^2^ value of 0.9999. In this work, MNC was only loaded into the tips of MNs as these microtips will penetrate the skin and deliver the drug. Baseplate was kept drug free as it will stay at the outer surface of the skin without penetrating the skin. It was essential to evaluate the ability of the reported MNs to load MNC. Our MNs had a MNC content of 673.06 ± 5.34 µg/array.

### 4.4. PLGA-Minocycline Tipped Microneedles In Vitro and Ex Vivo Dissolution Time

This study investigated the required time MNs tips to dissolve in full thickness rat skin. Tips started to dissolve immediately after 30 s of application into full thickness rat skin and they continued to dissolve gradually until 120 s were they completely dissolved as shown in Figure 4. This experiment was performed to estimate the wearing time. PLGA-MNC MNs are intended to be applied on the skin so having such a short wearing time of 2 min might support patients’ acceptance and adherence to this new delivery system.

### 4.5. PLGA-Minocycline Tipped Microneedles Ex Vivo Skin Deposition Study

**Figure 5:**
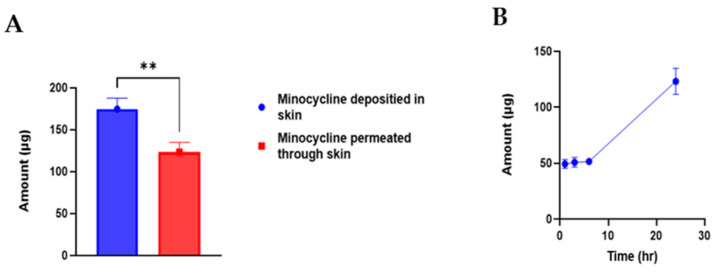
(**A**). Comparison between minocycline amount that was delivered into rat skin and minocycline amount that was permeated through rat skin after 24 h of PLGA-minocycline tipped microneedles upon performing ex vivo skin deposition study using Franz cells apparatus. (**B**). Amount of permeated minocycline into reservoir compartment over 24 h of PLGA-minocycline tipped microneedles upon performing ex vivo skin deposition study using Franz cells apparatus.

MNs technology has been intensively applied to deliver antibiotics topically into skin [45]. Targeted delivery of antibiotics enhances the desired therapeutic outcomes while minimizing the undesired systemic exposure. Ex vivo skin deposition study was performed to evaluate the ability of MNs to topically deliver MNC with minimum systemic exposure. Permeated amount of MNC through skin layers at predetermined timepoints were measured using UV-spectrophotometer. After 1 h of MNs application into Franz diffusion cell apparatus about 49.35 ± 4.02 µg of MNC was detected as shown in Figure 5. Followed by gradual increase in permeated amount after 3 and 6 h to be 50.76 ± 4.48 and 51.62 ± 1.83 µg. After 24 h, the total amount of permeated minocycline was 123.24 ± 11.62 µg which represents a percentage of 18.31% of the total loaded minocycline into MNs array. In terms of skin deposition, after 24 h of MNs application minocycline content in skin was analysed to be 174.64 ± 12.89 µg which represents a percentage of 26% of the total loaded MNC into MNs array. MNs significantly (*p* value = 0.0068) delivered MNC into skin more than into reservoir compartment demonstrating the ability of MNs to achieve targeted topical delivery of MNC. These findings align with the aim of this study and highlights the importance of applying MNs technology in antibiotics topical delivery.

### 4.6. PLGA-Minocycline Tipped Microneedles Antibacterial Potency

#### 4.6.1. Agar Diffusion Method

**Figure 6:**
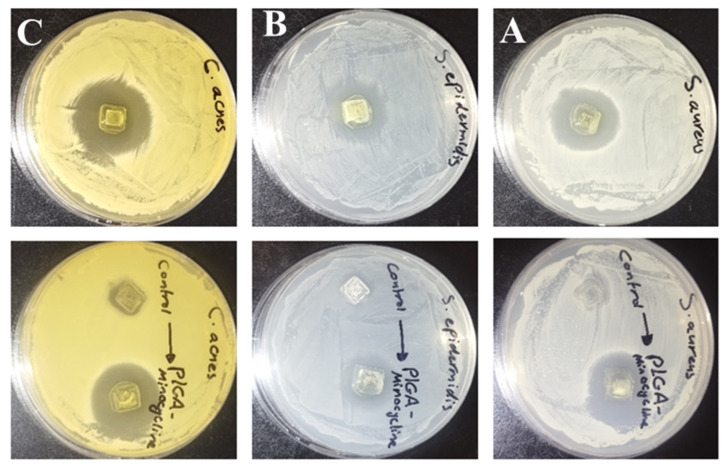
The antibacterial activity of PLGA-minocycline tipped microneedles and control microneedles (PLGA-DMSO) against (**A**) *S. aureus*, (**B**) *S. epidermidis*, and (**C**) *C. acnes* using agar diffusion method.

In order to evaluate the antibacterial activity of PLGA-MNC tipped MNs the agar diffusion method was used and compared with PLGA-DMSO tipped needle. The activity was tested against two Staphylococci species and C. acnes. As shown in Table 1 and Figure 6 the antibacterial activity of MNC was not affected during microneedle preparation and an inhibitory activity was recorded against all tested strains with highest activity was recorded against C. acnes where the inhibition zone was 3.1 cm.

#### 4.6.2. Minimum Inhibitory Concentration (MIC)

In order to examine the efficiency of prepared MNs against the tested bacterial species the MIC was determined for MNC alone, MNC -PLGA-DMSO (the microneedle tip solution) and as control the PLGA-DMSO solution alone. As shown in Table 2 the MIC values of MNC was not negatively affected during microneedle preparation where the MIC of both solutions were <0.146 µg/mL against all tested bacteria. This finding highly validates the preparation of MNC microneedle for topical application

#### 4.6.3. Minimum Bactericidal Concentration (MBC)

The MBC values was examined to determine the minimum concentration required for killing the tested bacteria. The lowest bactericidal activity of MNC was recorded against *S. aureus* (9.375 µg/mL) while the MBC against *S. epidermidis* and *C. acnes* was 18.75 µg/mL as Shown in Table 3.

## 5. Discussion

This work shows the successful formations and characterization of PLGA MNs loaded with MNC for innovative topical acne treatment. The carried-out studies reported that MNs demonstrated robust mechanical properties, antibacterial activity and skin insertion ability. These findings suggest that the reported MNs represent a promising alternative for topical MNC delivery for acne management. PLGA has been previously used to prepare MNs due to its suitable mechanical properties. The previously reported PLGA MNs had a sufficient strength to penetrate the skin to deliver therapeutics into the viable epidermis [25]. In alignment, PLGA-MNC MNs were fully formed and they were inserted into rat skin showing no mechanical failure. Upon compression force test, our MNs showed only 9.5% height reduction which confirms their mechanical acceptance. The mechanical strength and insertion ability might be the fundamental features of MNs. The basic drug delivery principle of MNs is to bypass the *stratum corneum* to overcome the limited permeation associated with the transdermal drug delivery route [42]. Further, our MNs had 673.06 ± 5.34 µg of MNC per array which is considered a high drug loading value compared to other previously reported MNs for antibiotic delivery [43]. Importantly, the reported MNs in this study showed a rapid dissolution. Our PLGA MNs completely dissolved within 2 min of application in ex vivo setups. MNs fabricated from other polymers such as hyaluronic acid might require much longer time to dissolve [43]. On the other hand, MNs made of highly hydrophilic polymers such as require about 10 s to dissolve but usually they lack the mechanical robustness [44]. PLGA was able to achieve a balance between mechanical robustness and acceptable dissolution time. Investigating the dissolution time is an essential step to estimate the required application time. Short application time might enhance user compliance and support their adherence to the treatment. In terms of the delivery efficiency of the reported MNs, 26% of the loaded MNC was delivered into skin while 18.3% was permeated through skin into receiver compartment in Franz cells apparatus. MNC is intended to be delivered into the *pilosebaceous* units where the bacteria colonise. Our findings support that MNs might give superior MNC delivery to the site of infection compared to other topical formulations. Evaluating the antibacterial potency of our reported MNs is a crucial step as well. Antibacterial experiments were carried out to confirm the preserved antibacterial activity of MNC after MNs formulation. MIC values were <0.146 µg/mL and MBC values were ranging from 9.375 to 18.75 µg/mL against *C. acnes*, *S. aureus* and *S. epidermis*. These findings support the effective use of our MNs in acne treatment as the tested bacteria types are implicated in acne pathophysiology. Overall, these findings support that MNC -PLGA MNs represent a promising innovative delivery system in acne treatment. This drug delivery system aims to overcome major challenges associated with the systemic and conventional topical delivery including limited MNC penetration through skin layers and systemic side effects with the oral administration. Furthermore, PLGA is FDA approved biodegradable and biocompatible polymer positioning it as an attractive polymer for safe topical delivery [45]. However, further investigations including intensive in vivo studies to evaluate the safety and efficacy of the reported MNs are essential prior transferring this technology into clinical practice.

## 6. Conclusions

The reported work in this study shows the successful formation and characterisation of PLGA MNC MNs for acne management. MNs demonstrated the required mechanical properties with high drug loading. Further, in vitro and ex vivo studies confirmed rapid dissolution and enhanced MNC deposition in skin layers. These findings support the potential of MNs to improve local drug delivery. Additionally, antibacterial studies confirmed the therapeutic relevance of our MNs on acne causing pathogens. In summary, the carried out studies suggest the suitability of PLGA MNC MNs as an innovative drug delivery system for minimally invasive and patient friendly local acne therapy. This innovative delivery system of MNC has the potential to support acne therapy by enhancing topical drug penetration into the pilosebaceous units within skin layers to minimize systemic exposure and adverse effects. Future studies will explore stability, expanded antimicrobial experiments, in vivo safety and efficacy to facilitate the translational potential of MNC MNs.

## Figures and Tables

**Table 1:** The antibacterial activity of PLGA-Minocycline MNs and control MNs (PLGA-DMSO) against *S. aureus*, *S. epidermidis*, and *C. acnes* using agar diffusion method. The diameter of inhibition zone was measured in cm ± SD.

Microneedles	* S. aureus * (ATCC 9144)	* S. epidermidis * (ATCC 51625)	* C. acnes * (ATCC 11827)
PLGA-Minocycline MNs	2.8 ± 0.11	2.3 ± 0.07	3.1 ± 0.11
Control MNs (PLGA-DMSO)	No inhibition	No inhibition	Slight inhibition

**Table 2:** MIC values of microneedles tips solution (minocycline-PLGA-DMSO), control 1 (minocycline solution) and control 2 (PLGA in DMSO solution) against *S. aureus*, *S. epidermidis*, and *C. acnes*.

Samples	* S. aureus * (ATCC 9144)	* S. epidermidis * (ATCC 51625)	* C. acnes * (ATCC 11827)
Minocycline-PLGA-DMSO solution	<0.146 µg/mL	<0.146 µg/mL	<0.146 µg/mL
Minocycline solution	<0.146 µg/mL	<0.146 µg/mL	<0.146 µg/mL
PLGA-DMSO solution	150 µg/mL	75 µg/mL	75 µg/mL

**Table 3:** MBC values of microneedles tips solution (minocycline-PLGA-DMSO), control 1 (minocycline solution) and control 2 (PLGA in DMSO solution) against *S. aureus*, *S. epidermidis*, and *C. acnes*.

Sample	* S. aureus * (ATCC 9144)	* S. epidermidis * (ATCC 51625)	* C. acnes * (ATCC 11827)
Minocycline-PLGA-DMSO solution	9.375 µg/mL	18.75 µg/mL	18.75 µg/mL
Minocycline solution	9.375 µg/mL	18.75 µg/mL	18.75 µg/mL
PLGA-DMSO solution	300 µg/mL	300 µg/mL	300 µg/mL

## Data Availability

The original contributions presented in this study are included in the article.

## Abbreviations

The following abbreviations are used in this manuscript:

MNs	Microneedles
MNC	Minocycline
PLGA	poly lactic-co-glycolic acid
UV	Ultra violet
DMSO	Dimethyl sulfoxide
